# Global assessment of select phytonutrient intakes by level of fruit and vegetable consumption

**DOI:** 10.1017/S0007114514001937

**Published:** 2014-08-11

**Authors:** Mary M. Murphy, Leila M. Barraj, Judith H. Spungen, Dena R. Herman, R. Keith Randolph

**Affiliations:** 1 Exponent, Inc., 1150 Connecticut Avenue, Northwest, Suite 1100, Washington, DC20036, USA; 2 Department of Family and Consumer Sciences, California State University, Northridge, CA91330, USA; 3 Nutrilite Health Institute, Buena Park, CA90621, USA

**Keywords:** Carotenoids, Flavonoids, Fruits, Vegetables, Dietary intake

## Abstract

Despite dietary recommendations that have repeatedly underscored the importance of increasing consumption of fruits and vegetables, intakes worldwide are lower than recommended levels. Consequently, the diets of many individuals may be lacking in nutrients and phytonutrients typical of a diet rich in a variety of fruits and vegetables. In the present study, we estimated phytonutrient intakes by adults categorised by sex, level of fruit and vegetable consumption ( < 5 *v.* ≥ 5 servings/d), and geographic diet cluster. Intakes of nine select phytonutrients were estimated from the 2002–4 World Health Survey fruit and vegetable servings intake data (*n* 198 637), the FAO supply utilisation accounts data, and phytonutrient concentration data obtained from the US Department of Agriculture databases and the published literature. Percentage contributions to each phytonutrient intake from fruit and vegetable sources were also estimated. Estimated intakes of phytonutrients from fruits and vegetables varied across the thirteen geographic diet clusters, reflecting regional differences in both numbers and proportions of fruit and vegetable servings consumed, and the specific types of fruits and vegetables available in the diet. The mean phytonutrient intakes by adults consuming ≥ 5 servings/d of fruits and vegetables were approximately 2- to 6-fold the mean phytonutrient intakes by adults with low fruit and vegetable consumption ( < 5 servings/d). In some cases, phytonutrient intakes by adults consuming ≥ 5 servings/d of fruits and vegetables in one geographic diet cluster were lower than the intakes by adults reporting < 5 servings/d in another cluster. The findings from this assessment provide important information regarding the major dietary patterns of phytonutrient intakes across geographic diet clusters.

Health and nutrition experts around the world have long recognised the importance of adequate intake of fruits and vegetables to support positive health outcomes. A body of evidence indicates that increased fruit and vegetable consumption is associated with a reduced risk of CVD, diabetes and stroke^(^ ^1^ ^–^ ^7^ ^)^. Fruits and vegetables are key sources of a number of essential nutrients, including K, Mg, dietary fibre, folate, and vitamins A and C^(^ ^8^ ^)^. They also contain an array of other bioactive substances, referred to as phytochemicals or phytonutrients^(^ ^8^ ^,^ ^9^ ^)^.

Studies have indicated that naturally occurring phytonutrients in fruits and vegetables may play important roles in health. For example, it has been suggested that β-carotene may be associated with a reduced risk of heart disease^(^ ^10^ ^)^. Similarly, dietary lycopene has been associated with a reduced risk of incident CVD^(^ ^11^ ^)^, while lutein and zeaxanthin may be instrumental in reducing the effects of oxidative injury that contribute to the development of age-related macular degeneration^(^ ^12^ ^)^. Ellagic acid, found in raspberries and strawberries, may reduce oxidative damage to DNA^(^ ^13^ ^,^ ^14^ ^)^. Consumption of anthocyanidins, a subclass of flavonoids found in fruits such as blueberries and grapes, may support verbal memory performance in individuals with mild cognitive impairments^(^ ^15^ ^)^. The results from *in vitro* studies show that the flavonoid quercetin, found in apples and onions, has antioxidant effects that are important for the prevention of certain cancers^(^ ^16^ ^,^ ^17^ ^)^, while animal studies have shown a stimulatory effect of high levels of quercetin on bone formation^(^ ^18^ ^)^. Hesperidin (a glycoside of hesperetin) may support endothelium-dependent microvascular reactivity^(^ ^19^ ^)^.

Fruit and vegetable intakes worldwide are lower than recommended levels^(^ ^20^ ^,^ ^21^ ^)^, consequently the diets of many individuals may be lacking in essential nutrients as well as phytonutrients typical of a diet rich in a variety of fruits and vegetables. There is a growing but limited body of information regarding phytonutrient intakes by populations of adults^(^ ^22^ ^–^ ^31^ ^)^. The available estimates were generated using a variety of survey methodologies and phytonutrient concentration databases; therefore, it is difficult to make direct comparisons across geographical regions. Additionally, not all geographic regions are represented with the data available.

Recent research has shown that individuals in the USA and Korea consuming recommended levels of fruits and vegetables have significantly higher intakes of select phytonutrients than individuals failing to meet the dietary recommendations^(^ ^32^ ^,^ ^33^ ^)^. While the findings of these studies are not necessarily surprising, they provide important information on phytonutrient intakes by level of fruit and vegetable consumption as well as dietary sources of phytonutrients in these two countries. Given regional differences in the availability of fruits and vegetables, it is reasonable to expect differences in phytonutrient intakes by level of fruit and vegetable consumption across geographical regions other than the USA and Korea.

An assessment of phytonutrient intakes by adults throughout the world and by level of fruit and vegetable consumption would provide important information for researchers to examine diet and health associations. An increased understanding of diet and health associations is necessary to develop guidelines on prudent phytonutrient intakes. The objective of the present study was to assess intakes of select phytonutrients by level of fruit and vegetable consumption in geographical regions throughout the world. Contributions of specific foods to total phytonutrient intakes from fruits and vegetables were also examined.

## Materials and methods

### Study population

Participants in the World Health Survey (WHS), a cross-sectional study conducted between 2002 and 2004 in seventy countries, comprised the study population^(^ ^34^ ^)^. Using multi-stage cluster sampling, the WHS selected a nationally representative sample of adults aged 18 years and older, proportionately allocated into three to five strata defined by sex, socio-economic status, geography and potentially one or two additional strata (not identified in the survey documentation) from each country participating in the study. Data collected at the individual level in the WHS included sociodemographic information, health state descriptions, health state valuation, risk factors, chronic conditions, mortality, health care utilisation, health systems' responsiveness and social capital. The study population for the present analysis was limited to males and females aged 18 years and older in the WHS with responses to the questionnaire on fruit and vegetable consumption administered to individuals in a subset of participating countries (*n* 198 637). Reported intakes of fruits and vegetables ranged from 0 to 100 servings/d. The study sample was further restricted to individuals reporting 15 or fewer total servings of fruits and vegetables per d (corresponding to the 99th percentile of intake among adults with age, sex, and fruit and vegetable servings data), which resulted in the exclusion of 1712 participants; the total sample was 196 925 adults. Participation in the survey was voluntary, and interviewers obtained written consent from survey participants before the interview^(^ ^35^ ^)^.

### Levels of total fruit and vegetable consumption in the study population

Respondents in a total of fifty-two geographically diverse, though mainly low- and middle-income countries participating in the WHS were administered the ‘long’ version of the questionnaire, which included questions on fruit and vegetable consumption^(^ ^35^ ^)^. Specifically, participants were asked two questions: (1) How many servings of fruit do you eat on a typical day? and (2) How many servings of vegetables do you eat on a typical day? A ‘typical day’ was considered a day when an individual ate fruits or vegetables; respondents were advised not to estimate average consumption over time. Trained interviewers showed each respondent cards with examples of serving sizes for fruits and vegetables typically consumed in that country^(^ ^35^ ^)^. The available survey documentation notes that, in general, one serving of vegetables was considered to be one cup of raw green leafy vegetables such as spinach or salad; one-half cup of other vegetables cooked or chopped raw, such as tomatoes, carrots, pumpkin, maize, Chinese cabbage, fresh beans or onions; or one-half cup of vegetable juice. One serving of fruit was considered to be one medium-sized piece of fruit, such as an apple, banana or orange; one-half cup of chopped, cooked or canned fruit; or one-half cup of fruit juice. Information on typical consumption of other foods was not collected in the questionnaire; therefore, the present analysis was limited to phytonutrient intakes from fruits and vegetables.

### Fruits and vegetables available by geographical region

The WHS did not collect information on the specific types of fruits and vegetables consumed by each respondent. However, data from the WHO and the FAO provided quantitative information on the availability of specific types of fruits and vegetables by country; these data were used to estimate the proportions of fruits and vegetables available by type in each geographical region.

Beginning in the early 1990s, the WHO Global Environment Monitoring System/Food Contamination Monitoring and Assessment Programme (GEMS/Food) developed an approach for categorising countries into clusters with similar dietary intakes and calculating a representative diet for each cluster. The diets are based on per capita food availability data derived from annual food production, imports and exports statistics compiled by the FAO and released as FAO supply utilisation accounts^(^ ^36^ ^)^. The food availability data represent the total quantity of food available at the per capita level, including quantities produced and imported, adjusted by quantities exported, or quantities used as animal feed or for seed. These data provide a proxy for consumption rather than actual consumption. In 2006, the WHO used cluster analysis to group countries throughout the world into thirteen statistical clusters consisting of seven to twenty-two countries each, based on 1997–2001 FAO data and geographical proximity of the countries within the statistical clusters^(^ ^37^ ^)^. The WHO used a letter-coding system (A through M) to identify geographic diet clusters referred to as the GEMS/Food clusters (see online supplementary Fig. S1). In 2012, the WHO generated new cluster groupings and diets based on 2002–7 FAO data, though geographical proximity was not used to define the new clusters. The objective of the present study was to examine diets by geographical region. Therefore, we obtained the FAO supply utilisation accounts data from the developers of the 2012 cluster diets as these data provided the most current information on food availability^(^ ^38^ ^)^. Using these food availability data, we generated updated food consumption estimates for the thirteen regions defined by the 2006 geographic diet clusters by weighting each country's data with its relative population size and deriving weighted average consumption levels for each geographic diet cluster. For the present analysis, the FAO data were converted from kg/year to g/d.

The 2002–7 FAO data provided estimates of food availability for a total of 415 specific food categories or groupings of similar foods within a hierarchical system of eighteen broad food categories. A total of sixty-seven categories of fruits and forty-two categories of vegetables were identified for inclusion in the present analysis (see online supplementary Table S1). The fruit categories included juices and olives but excluded nuts and seeds. The vegetable categories included juices but excluded pulses, herbs, and roots and tubers such as potatoes and cassava. Some of the identified FAO food categories included the descriptor ‘nes’, the abbreviation for ‘not elsewhere specified’. The ‘nes’ categories were used by the FAO to capture the availability of foods not identified in a specific category, although it is likely that some countries used the ‘nes’ categories for reporting the availability of foods that were identified in a specific category, particularly if the foods were of limited local importance^(^ ^36^ ^,^ ^39^ ^)^. With the exception of the ‘Juice of Vegetables nes’ and ‘Fruit Prp nes’ categories, food intakes from fruits and vegetables classified as ‘nes’ were allocated to the corresponding fruit or vegetable categories in amounts proportionate to the relative availability of foods in each category within each geographic diet cluster. For example, intake of ‘Pome Fruit nes’ was allocated to intakes in the individual categories for ‘Apples’, ‘Pears’ and ‘Quinces’ based on the total intake of ‘Pome Fruit nes’ and the relative availability of each of the three categories within each geographic diet cluster. This approach is consistent with that used in previous analyses of food intakes using FAO data (A Vieira, personal communication)^(^ ^22^ ^)^. The reallocation of the ‘nes’ categories resulted in fifty-eight categories of fruits and forty categories of vegetables.

### Phytonutrient concentration data for categories of fruits and vegetables

Phytonutrient concentration data were identified for select carotenoids, flavonoids and a phenolic acid (ellagic acid) for the specific fruits and vegetables in each of the FAO categories for fruits and vegetables (see online supplementary Table S1 for a list of the foods included in each category). The nine phytonutrients included in the present analysis are found predominantly in fruits and vegetables and represent phytonutrients from major classes of phytochemicals. For the flavanone and flavonol subclasses of flavonoids, we estimated intakes of hesperetin and quercetin, respectively. Previous research on a Spanish cohort indicated that these two flavonoids account for the majority of dietary intake within their respective flavonoid subclasses^(^ ^40^ ^)^.

Carotenoid concentration data (α-carotene, β-carotene, β-cryptoxanthin, lutein/zeaxanthin and lycopene) were obtained from the US Department of Agriculture (USDA) National Nutrient Database for Standard Reference, Release 25^(^ ^41^ ^)^, which includes data compiled from published and unpublished sources. The USDA database did not include carotenoid values for two relevant food categories: cashew apple and cassava leaves. β-Carotene values for these foods were imputed from another data source^(^ ^42^ ^)^. Anthocyanidins (the sum of cyanidin, delphinidin, malvidin, pelargonidin, peonidin and petunidin), hesperetin and quercetin values (reported as mg aglycone/100 g edible portion) were obtained from the most recent release of the USDA's flavonoid database available at the time of the analysis^(^ ^43^ ^)^. The USDA database contains analytical flavonoid data systematically compiled from international sources. This database of flavonoid values has served as the sole or primary flavonoid concentration data source in numerous international analyses of phytonutrient intakes^(^ ^22^ ^–^ ^25^ ^,^ ^29^ ^)^. Missing flavonoid values for concentrated juices and dried foods were imputed from the available data for single-strength juices or the fresh form with adjustments for moisture. Missing flavonoid values for cooked foods were imputed from data on raw foods, assuming 25 % retention^(^ ^44^ ^)^.

The USDA databases do not contain concentration data for ellagic acid. Ellagic acid equivalent concentration data for foods in the FAO fruit and vegetable categories were obtained from a database of values compiled from the published literature^(^ ^45^ ^–^ ^58^ ^)^. The database of ellagic acid values included ellagic acid from all sources (i.e. free ellagic acid, ellagitannins and other sources) based on analysis after acid hydrolysis, with results reported as ellagic acid equivalents. Non-zero concentrations of ellagic acid were identified for seventeen specific categories of fruits included in the analysis.

Many of the FAO fruit and vegetable categories corresponded to a single item, for example spinach or bananas, though some categories encompassed multiple foods, for example the category ‘Cabbages and other brassicas’ or the category ‘Carrots and turnips’. Each FAO category was matched to the available phytonutrient data for the representative raw and/or cooked forms in which the food(s) would typically be consumed. In this analysis, the ‘Cabbages and other brassicas’ category included averages of the available phytonutrient concentration data for cabbages, Brussels sprouts, collards, cress, kale, kohlrabi, mustard greens, radish, rutabagas and turnip greens^(^ ^59^ ^)^. In the absence of more detailed data on the consumption of specific foods within these broad categories, the available phytonutrient concentration data were averaged to create a representative phytonutrient concentration profile (phytonutrient amount/100 g food as consumed) for each FAO category. In some cases, the available anthocyanidin concentration data varied considerably across fruits or vegetables included within a category. For example, red grapes, a type of grape included in the ‘Grapes’ category, contain relatively high levels of anthocyanidins^(^ ^43^ ^)^. In contrast, no anthocyanidin concentration data are reported for green grapes (also included in the ‘Grapes’ category) in the USDA flavonoid database, though they are presumably not a significant source of this flavonoid^(^ ^60^ ^)^. To allow for a more representative average anthocyanidin concentration, we assumed a concentration of zero for items with missing anthocyanidin values (e.g. green grapes).

### Defining low fruit and vegetable consumption

The WHO panel on diet, nutrition and prevention of chronic diseases recommended a daily intake of at least 400 g of fruits and vegetables, excluding potatoes, cassava and other tubers^(^ ^61^ ^)^. Based on the WHO recommendation, an intake of < 400 g of fruits and vegetables, or a minimum of 5 servings daily with an average serving size of 80 g, has been characterised as low consumption of fruits and vegetables^(^ ^20^ ^)^.

### Statistical analysis

Data for WHS respondents were grouped based on the geographic diet cluster to which their country of residence was assigned. Daily intakes of fruits, vegetables, and fruits and vegetables combined were estimated for males and females categorised by level of total fruit and vegetable consumption ( < 5 or ≥ 5 servings/d) in each geographic diet cluster.

Percentage contributions of each category of fruits and vegetables by weight to the total availability of fruits and vegetables were calculated at the population level in each geographic diet cluster. Percentage contributions were derived by dividing the per capita availability in each fruit and each vegetable category by the per capita availability summed separately across all categories of fruits and all categories of vegetables, respectively.

For each geographic diet cluster, availability (in g/d) of each category of fruit and each category of vegetables was multiplied by the concentration of each phytonutrient in the category; these values were summed to estimate total daily phytonutrient intakes from fruits and from vegetables in the cluster. Since the WHO/FAO have recommended a daily intake of at least 400 g of fruits and vegetables, and have also recommended daily consumption of at least 5 servings of fruits and vegetables, it was assumed that the average size of the fruit and vegetable serving was 80 g. For each cluster, the total estimated phytonutrient intakes from fruits and from vegetables were divided by the total gram intake of each category (i.e. fruits or vegetables) and multiplied by eighty to derive phytonutrient contents per serving of fruits and per serving of vegetables. Using the calculated phytonutrient values per serving of fruits and vegetables and the reported numbers of fruit and vegetable servings for each WHS respondent, estimated average daily intakes of phytonutrients were calculated by geographic diet cluster, sex, and level of fruit and vegetable consumption.

In each geographic diet cluster, ranked contributions of each fruit and vegetable category to total phytonutrient intakes were calculated for the population of adults. Ranked contributions were calculated as the proportion of phytonutrient intakes from each fruit and vegetable category relative to the total phytonutrient intakes from all fruits and vegetables combined. Contributions from similar categories were combined into a single category (e.g. the ‘Single-strength orange juice’ and ‘Concentrated orange juice’ categories were combined and reported as ‘Orange juice’; the ‘Tomatoes’ and ‘Tomato peeled’ categories were combined and reported as ‘Tomatoes’) before ranking the categories. The ranked phytonutrient contributions were summarised for fifty-one categories of fruits and thirty-four categories of vegetables.

The WHO released sampling weights at the individual level to adjust for survey design and non-response, though the weights were not available for adults in all fifty-two countries included in the present analysis. Hence, the WHO sampling weights were not used in the analysis. Alternative sampling weights were assigned to the WHS participants to reflect the number of participants in each country and the relative proportion of adults in each country to all countries in the cluster; these alternative sampling weights were used in all analyses.

## Results

### Servings of fruits and vegetables by geographic diet cluster and level of fruit and vegetable consumption

The mean daily intakes of fruits and vegetables, and fruits and vegetables combined by geographic diet cluster and overall level of fruit and vegetable consumption ( < 5 (low) *v.* ≥ 5 servings/d) are shown in Table 1. Across all geographic diet clusters, the majority of adults (58–88 %) reported low consumption of fruits and vegetables. In the subpopulation of adults with low fruit and vegetable consumption, daily intakes of fruits ranged from 0·7 to 1·5 servings across the geographic diet clusters and those of vegetables ranged from 0·9 to 1·6 servings; combined daily intakes of fruits and vegetables ranged from 1·6 to 2·8 servings. Among the subpopulation of adults with daily intakes of ≥ 5 servings of fruits and vegetables, the mean daily intakes of fruits ranged from 2·5 to 4·8 servings and those of vegetables ranged from 2·1 to 4·0 servings. The mean combined daily intakes of fruits and vegetables ranged from 6·0 to 7·6 servings.

**Table 1:** Estimated daily intakes of fruits (F) and vegetables (V), and fruits and vegetables combined (F&V) in the 2002–4 World Health Survey (WHS) assessed by geographic diet cluster (Number of participants, percentages, and mean values with their standard errors)

			< 5 F&V servings/d	≥ 5 F&V servings/d
			*n*	%	F	V	F&V	*n*	%	F	V	F&V
Sex, geographic diet cluster*	Mean	se	Mean	se	Mean	se	Mean	se	Mean	se	Mean	se
Men																		
	M	Americas and Australia	1082	75	1·2	0·03	1·2	0·02	2·4	0·04	358	25	3·6	0·09	2·9	0·07	6·5	0·10
	H	South/Central America	2801	72	1·3	0·02	1·0	0·01	2·2	0·03	1249	28	4·8	0·07	2·1	0·05	6·8	0·06
	K	South America	2816	58	1·0	0·05	1·1	0·05	2·2	0·08	971	42	3·5	0·12	3·5	0·13	7·0	0·15
	B	Southern Europe/Mediterranean	5956	78	1·4	0·01	1·2	0·01	2·7	0·01	1648	22	3·8	0·04	2·3	0·03	6·1	0·04
	E	Western Europe	1952	87	1·2	0·02	1·1	0·01	2·3	0·02	291	13	3·6	0·10	2·7	0·09	6·3	0·10
	F	Northern Europe	515	78	1·1	0·03	1·4	0·03	2·4	0·04	132	22	3·0	0·13	3·5	0·11	6·6	0·16
	D	Eastern Europe	4617	81	0·9	0·01	1·4	0·01	2·4	0·02	1015	19	3·1	0·06	3·8	0·06	6·9	0·10
	G	Asia†	22 676	85	0·8	0·01	1·6	0·01	2·4	0·01	4790	15	2·5	0·04	3·6	0·04	6·2	0·04
	L	Asia†	3513	76	1·3	0·01	1·4	0·01	2·7	0·02	1129	24	3·8	0·06	2·6	0·04	6·4	0·06
	C	Northern Africa/Middle East	3878	85	0·7	0·01	1·6	0·02	2·3	0·02	492	15	2·5	0·09	4·0	0·09	6·4	0·08
	A	Central Africa‡	3982	64	1·1	0·03	1·1	0·03	2·2	0·05	1262	36	3·9	0·10	3·2	0·07	7·1	0·09
	J	Central Africa‡	6184	77	0·7	0·01	0·9	0·01	1·7	0·02	1736	23	4·5	0·07	3·2	0·06	7·6	0·07
	I	Southern Africa	8427	69	1·0	0·01	1·4	0·01	2·4	0·02	3789	31	4·2	0·05	3·0	0·04	7·2	0·06
Women																		
	M	Americas and Australia	1055	69	1·4	0·03	1·2	0·02	2·6	0·04	472	31	3·5	0·06	2·8	0·06	6·3	0·07
	H	South/Central America	4204	78	1·3	0·02	1·0	0·01	2·3	0·02	1334	22	4·1	0·06	2·3	0·05	6·5	0·05
	K	South America	3904	61	1·3	0·04	1·2	0·04	2·5	0·06	1022	39	3·3	0·09	3·5	0·10	6·9	0·11
	B	Southern Europe/Mediterranean	8035	77	1·5	0·01	1·2	0·01	2·7	0·01	2219	23	3·8	0·03	2·2	0·02	6·0	0·03
	E	Western Europe	2765	81	1·3	0·02	1·2	0·01	2·5	0·02	612	19	3·7	0·08	2·7	0·06	6·4	0·09
	F	Northern Europe	949	79	1·2	0·02	1·4	0·02	2·5	0·03	255	21	3·1	0·09	3·4	0·08	6·5	0·11
	D	Eastern Europe	7737	79	1·0	0·01	1·4	0·01	2·4	0·02	1873	21	3·3	0·04	3·6	0·04	6·8	0·06
	G	Asia†	25 784	86	0·8	0·01	1·6	0·01	2·4	0·01	5362	14	2·5	0·04	3·7	0·04	6·1	0·04
	L	Asia†	4166	77	1·4	0·01	1·3	0·01	2·8	0·01	1241	23	3·7	0·05	2·6	0·04	6·3	0·05
	C	Northern Africa/Middle East	5049	88	0·7	0·01	1·5	0·01	2·2	0·02	555	12	2·7	0·08	3·4	0·07	6·0	0·07
	A	Central Africa‡	3990	66	1·0	0·03	1·2	0·03	2·2	0·05	1232	34	3·5	0·08	3·1	0·06	6·6	0·08
	J	Central Africa‡	7381	79	0·7	0·01	1·0	0·01	1·6	0·02	1687	21	4·1	0·07	3·3	0·06	7·4	0·07
	I	Southern Africa	11 642	69	1·1	0·01	1·5	0·01	2·5	0·02	5139	31	4·0	0·04	2·9	0·03	6·9	0·05

* Geographic diet clusters were based on the 2006 Global Environment Monitoring System (GEMS)/Food Contamination Monitoring and Assessment Programme (GEMS/Food) clusters and the 2002–7 FAO supply utilisation accounts data; fruit and vegetable intakes were as reported in the 2002–4 WHS data.

† Asia was separated by the GEMS into two clusters; both diets were high in rice and wheat. Cluster G was characterised by higher availability of fruiting vegetables, milk and milk products, potatoes, and fish/seafood and fish/seafood products, while cluster L was characterised by higher availability of fish/seafood and fish/seafood products, maize, milk and milk products, and brassica vegetables.

‡ Central Africa was separated by the GEMS into two clusters. Cluster A was characterised by higher availability of plantains, cassava, rice, wheat, maize, and milk and milk products. Cluster J was characterised by higher availability of cassava, sorghum, milk and milk products, millet, rice, and maize.

### Availability of fruits and vegetables by geographic diet cluster

**Fig. 1:**
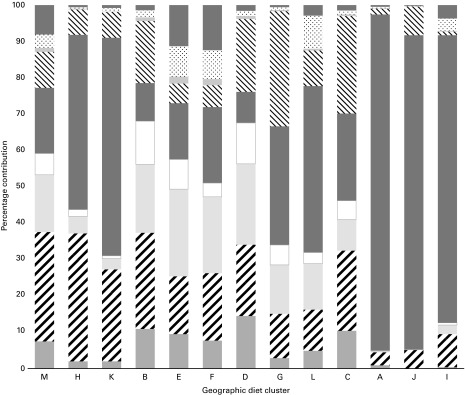
Percentage contributions of total fruits by type in the thirteen geographic diet clusters. Geographic diet clusters were based on the 2006 Global Environment Monitoring System (GEMS)/Food Contamination Monitoring and Assessment Programme (GEMS/Food) clusters and the 2002–7 FAO supply utilisation accounts data. M, Americas and Australia; H, South/Central America; K, South America; B, Southern Europe/Mediterranean; E, Western Europe; F, Northern Europe; D, Eastern Europe; G, Asia; L, Asia; C, Northern Africa/Middle East; A, Central Africa; J, Central Africa; I, Southern Africa. Asia was separated by the GEMS into two clusters; both diets were high in rice and wheat. Cluster G was characterised by higher availability of fruiting vegetables, milk and milk products, potatoes, and fish/seafood and fish/seafood products, while cluster L was characterised by higher availability of fish/seafood and fish/seafood products, maize, milk and milk products, and brassica vegetables. Central Africa was separated by the GEMS into two clusters. Cluster A was characterised by higher availability of plantains, cassava, rice, wheat, maize, and milk and milk products. Cluster J was characterised by higher availability of cassava, sorghum, milk and milk products, millet, rice, and maize. Values are mean percentages; the percentage of total fruits available from the ‘Fruit/vegetable juices’ category reflects the contribution from fruit juices. 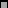, Berries/other small fruits; 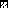, citrus fruits; 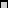, pome fruits; 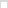, stone fruits; 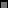, tropical/subtropical fruits; 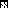, watermelons, other melons; 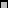, dried fruits; 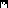, prepared fruits (not dried, juice); 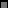, fruit/vegetable juices.

**Fig. 2:**
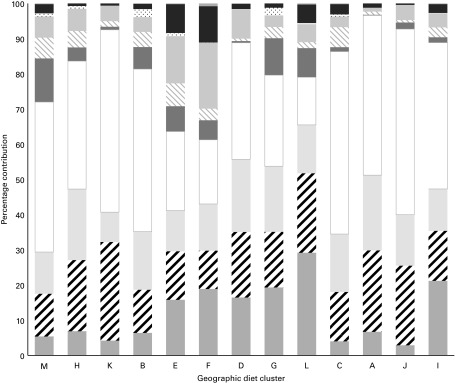
Percentage contributions of total vegetables by type in the thirteen geographic diet clusters. Geographic diet clusters were based on the 2006 Global Environment Monitoring System (GEMS)/Food Contamination Monitoring and Assessment Programme (GEMS/Food) clusters and the 2002–7 FAO supply utilisation accounts data. M, Americas and Australia; H, South/Central America; K, South America; B, Southern Europe/Mediterranean; E, Western Europe; F, Northern Europe; D, Eastern Europe; G, Asia; L, Asia; C, Northern Africa/Middle East; A, Central Africa; J, Central Africa; I, Southern Africa. Asia was separated by the GEMS into two clusters; both diets were high in rice and wheat. Cluster G was characterised by higher availability of fruiting vegetables, milk and milk products, potatoes, and fish/seafood and fish/seafood products, while cluster L was characterised by higher availability of fish/seafood and fish/seafood products, maize, milk and milk products, and brassica vegetables. Central Africa was separated by the GEMS into two clusters. Cluster A was characterised by higher availability of plantains, cassava, rice, wheat, maize, and milk and milk products. Cluster J was characterised by higher availability of cassava, sorghum, milk and milk products, millet, rice, and maize. Values are mean percentages; the percentage of total vegetables available from the ‘Fruit/vegetable juices’ category reflects the contribution from vegetable juices. 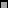, Brassica vegetables; 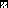, bulb vegetables; 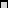, fruiting vegetables (cucurbits); 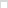, fruiting vegetables (not cucurbits)/mushrooms; 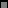, leafy vegetables; 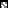, legume vegetables; 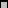, root vegetables; 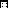, stalk/stem vegetables; 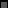, other/mixed vegetables; 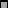, fruit/vegetable juices.

Availability of the specific categories of fruits and vegetables was summarised as nine types of fruits and ten types of vegetables. Percentage contributions (by weight) to total fruits and vegetables available by type showed regional differences in the relative proportions of different types of fruits and vegetables available in the diet (Figs. 1 and 2). Tropical/subtropical fruits (including bananas and plantains) were the predominant fruits available in several regions including Central and Southern Africa, South America, and Asia. In the remaining geographic diet clusters, citrus fruits, pome fruits, or watermelons and other melons accounted for the largest proportion of fruits. Fruiting vegetables (excluding cucurbits) and mushrooms, the vegetable category that includes tomatoes, accounted for 33 % or more of the total vegetables in all geographic diet clusters other than clusters in Asia and western and northern regions of Europe.

### Phytonutrient intakes by geographic diet cluster and level of fruit and vegetable consumption

The estimates of carotenoid intakes by geographic diet cluster and level of fruit and vegetable consumption are presented in Table 2, and the estimates of select flavonoid and ellagic acid intakes are summarised in Table 3. Across both men and women, the mean phytonutrient intakes by adults consuming ≥ 5 servings/d of fruits and vegetables were approximately 2- to 6-fold the mean phytonutrient intakes by those with low fruit and vegetable consumption.

**Table 2:** Estimated daily intakes of carotenoids (μg/d) from fruits and vegetables (F&V) by level of combined F&V consumption in the 2002–4 World Health Survey (WHS) assessed by geographic diet cluster (Mean values with their standard errors)

			α-Carotene (μg/d)	β-Carotene (μg/d)	β-Cryptoxanthin (μg/d)	Lutein/zeaxanthin (μg/d)	Lycopene (μg/d)
			< 5 F&V servings/d	≥ 5 F&V servings/d	< 5 F&V servings/d	≥ 5 F&V servings/d	< 5 F&V servings/d	≥ 5 F&V servings/d	< 5 F&V servings/d	≥ 5 F&V servings/d	< 5 F&V servings/d	≥ 5 F&V servings/d
Sex, geographic diet cluster*	Mean	se	Mean	se	Mean	se	Mean	se	Mean	se	Mean	se	Mean	se	Mean	se	Mean	se	Mean	se
Men																						
	M	Americas and Australia	204	3·7	498	11·3	1137	20·2	2799	60·5	72	1·5	213	4·1	991	17·9	2425	54·3	1235	20·2	3153	55·5
	H	South/Central America	210	2·6	526	7·0	825	10·4	2028	28·4	101	1·3	339	4·0	555	7·4	1286	22·1	1170	13·7	3372	31·5
	K	South America	190	7·5	596	15·4	719	29·5	2229	63·3	72	3·2	245	7·7	390	16·6	1201	37·1	1714	68·7	5352	142·5
	B	Southern Europe/Mediterranean	162	0·9	310	3·6	997	5·2	1986	19·4	87	0·6	220	1·8	764	4·1	1490	16·1	1740	8·4	3681	27·5
	E	Western Europe	352	4·2	858	26·1	1570	18·4	3862	111·4	67	0·9	195	4·4	1261	15·0	3090	91·5	642	6·8	1640	35·7
	F	Northern Europe	560	12·2	1462	45·5	2187	47·1	5710	175·5	72	1·6	201	6·7	1545	33·2	4037	123·5	784	14·8	2085	53·1
	D	Eastern Europe	304	3·0	796	12·6	1375	13·2	3668	55·0	59	0·7	186	3·1	1066	10·5	2811	43·6	1585	14·5	4508	61·6
	G	Asia†	163	1·0	375	3·2	1563	9·1	3627	28·7	70	0·5	204	2·4	1897	11·8	4286	40·5	1460	10·1	4109	41·1
	L	Asia†	219	1·6	444	5·2	1728	12·7	3491	41·5	121	1·0	332	4·5	2092	16·1	4138	54·2	788	5·3	2011	21·6
	C	Northern Africa/Middle East	185	1·9	473	10·4	891	8·5	2350	42·2	47	0·7	151	3·7	599	5·9	1552	31·2	2132	19·8	5907	80·3
	A	Central Africa‡	291	6·5	973	15·0	697	15·7	2179	29·6	45	1·0	140	1·9	627	15·5	1830	38·9	794	18·2	2432	36·6
	J	Central Africa‡	182	2·2	828	8·0	618	7·3	2537	26·8	63	0·9	328	4·0	345	4·4	1219	19·9	1077	13·2	5095	51·6
	I	Southern Africa	287	2·3	829	7·1	1366	11·4	3192	35·1	45	0·4	133	1·1	1462	13·2	3122	41·6	1190	9·7	2883	29·6
Women																						
	M	Americas and Australia	213	3·5	498	9·9	1191	18·8	2775	52·7	82	1·4	207	2·9	1036	16·6	2407	47·3	1315	18·7	3113	48·2
	H	South/Central America	217	2·2	548	7·3	852	8·6	2133	29·7	102	1·0	308	3·0	574	6·1	1393	22·9	1198	11·1	3287	29·2
	K	South America	212	5·0	595	12·3	793	19·6	2236	51·3	89	2·5	233	5·7	427	11·1	1210	30·3	1905	45·6	5350	114·6
	B	Southern Europe/Mediterranean	162	0·8	297	2·8	1001	4·3	1909	14·7	89	0·5	219	1·5	766	3·4	1428	12·1	1756	7·0	3570	21·1
	E	Western Europe	366	3·5	852	18·3	1636	15·3	3843	78·4	72	0·7	201	3·3	1312	12·4	3071	64·2	674	5·7	1647	26·6
	F	Northern Europe	561	8·4	1403	32·4	2191	32·3	5482	125·1	76	1·1	203	4·4	1549	22·8	3877	88·0	798	10·3	2031	37·0
	D	Eastern Europe	299	2·2	754	8·2	1364	9·5	3503	35·7	62	0·5	190	2·2	1053	7·5	2670	28·3	1606	10·3	4426	41·0
	G	Asia†	162	0·9	376	3·2	1550	8·3	3637	29·0	73	0·5	203	2·2	1873	10·8	4305	40·1	1498	9·5	4082	37·2
	L	Asia†	217	1·3	436	5·3	1709	10·7	3432	42·4	129	0·9	326	3·9	2060	13·7	4069	55·3	824	4·7	1977	18·3
	C	Northern Africa/Middle East	179	1·5	402	7·7	864	7·0	2059	31·0	45	0·6	155	3·4	581	4·8	1338	23·1	2065	16·6	5413	61·6
	A	Central Africa‡	295	6·4	899	13·4	727	15·7	2065	25·7	46	1·0	132	1·6	672	15·9	1781	33·3	835	18·4	2323	31·5
	J	Central Africa‡	179	2·0	807	7·7	619	6·9	2537	26·1	60	0·8	310	3·8	355	4·3	1270	19·7	1047	12·2	4918	49·1
	I	Southern Africa	297	1·9	795	5·2	1414	9·4	3081	25·6	47	0·3	127	0·8	1514	10·8	3023	30·8	1232	8·0	2779	21·4

* Geographic diet clusters were based on the 2006 Global Environment Monitoring System (GEMS)/Food Contamination Monitoring and Assessment Programme (GEMS/Food) clusters and the 2002–7 FAO supply utilisation accounts data; fruit and vegetable intakes were as reported in the 2002–4 WHS data; carotenoid concentration data were obtained from the US Department of Agriculture National Nutrient Database, Standard Reference Release 25.

† Asia was separated by the GEMS into two clusters; both diets were high in rice and wheat. Cluster G was characterised by higher availability of fruiting vegetables, milk and milk products, potatoes, and fish/seafood and fish/seafood products, while cluster L was characterised by higher availability of fish/seafood and fish/seafood products, maize, milk and milk products, and brassica vegetables.

‡ Central Africa was separated by the GEMS into two clusters. Cluster A was characterised by higher availability of plantains, cassava, rice, wheat, maize, and milk and milk products. Cluster J was characterised by higher availability of cassava, sorghum, milk and milk products, millet, rice, and maize.

**Table 3:** Estimated daily intakes of select flavonoids and ellagic acid (mg/d) from fruits and vegetables (F&V) by level of combined F&V consumption in the 2002–4 World Health Survey (WHS) assessed by geographic diet cluster (Mean values with their standard errors)

			Anthocyanidins (mg/d)	Hesperetin (mg/d)	Quercetin (mg/d)	Ellagic acid (mg/d)
			< 5 F&V servings/d	≥ 5 F&V servings/d	< 5 F&V servings/d	≥ 5 F&V servings/d	< 5 F&V servings/d	≥ 5 F&V servings/d	< 5 F&V servings/d	≥ 5 F&V servings/d
Sex, geographic diet cluster*	Mean	se	Mean	se	Mean	se	Mean	se	Mean	se	Mean	se	Mean	se	Mean	se
Men																		
	M	Americas and Australia	7·0	0·13	20·1	0·34	8·0	0·20	24·9	0·61	4·7	0·08	12·0	0·22	2·2	0·06	7·0	0·17
	H	South/Central America	2·6	0·03	8·3	0·08	9·6	0·15	35·8	0·54	5·0	0·07	11·3	0·21	0·7	0·01	2·5	0·04
	K	South America	2·8	0·11	9·4	0·23	5·1	0·24	17·6	0·60	5·5	0·24	16·6	0·57	0·3	0·01	0·9	0·03
	B	Southern Europe/Mediterranean	5·6	0·03	13·9	0·10	7·5	0·06	20·0	0·19	4·7	0·02	9·4	0·09	1·2	0·01	3·1	0·03
	E	Western Europe	6·4	0·08	18·4	0·39	4·1	0·06	12·5	0·36	4·2	0·04	10·6	0·23	2·5	0·04	7·6	0·22
	F	Northern Europe	5·8	0·12	16·1	0·50	4·3	0·11	12·1	0·51	4·1	0·08	10·9	0·29	2·0	0·05	5·8	0·24
	D	Eastern Europe	5·9	0·07	18·7	0·32	3·5	0·05	11·7	0·23	5·9	0·06	15·9	0·23	0·8	0·01	2·6	0·05
	G	Asia†	3·5	0·02	9·1	0·06	1·4	0·01	4·4	0·07	5·8	0·03	13·3	0·11	0·2	0·00	0·6	0·01
	L	Asia†	6·2	0·04	15·9	0·18	1·5	0·01	4·3	0·07	5·8	0·04	11·8	0·14	1·9	0·02	5·6	0·09
	C	Northern Africa/Middle East	2·7	0·04	8·8	0·22	3·0	0·06	10·6	0·37	4·9	0·05	12·7	0·25	0·4	0·01	1·2	0·04
	A	Central Africa‡	2·3	0·05	8·0	0·14	0·8	0·02	2·8	0·07	4·5	0·11	13·1	0·29	0·1	0·00	0·4	0·01
	J	Central Africa‡	1·0	0·01	4·3	0·04	0·7	0·01	4·4	0·07	5·0	0·06	17·2	0·30	0·1	0·00	0·5	0·01
	I	Southern Africa	3·4	0·03	11·2	0·10	2·0	0·03	8·3	0·10	5·5	0·05	11·8	0·15	0·2	0·00	0·8	0·01
Women																		
	M	Americas and Australia	7·8	0·12	19·5	0·24	9·4	0·19	24·1	0·42	5·0	0·07	11·8	0·20	2·6	0·05	6·7	0·12
	H	South/Central America	2·7	0·03	7·8	0·07	9·6	0·11	31·3	0·42	5·2	0·06	12·4	0·22	0·7	0·01	2·2	0·03
	K	South America	3·4	0·08	9·0	0·16	6·4	0·19	16·7	0·45	5·9	0·16	16·8	0·47	0·3	0·01	0·9	0·02
	B	Southern Europe/Mediterranean	5·7	0·03	13·8	0·09	7·7	0·05	19·9	0·16	4·7	0·02	9·0	0·07	1·2	0·01	3·1	0·02
	E	Western Europe	6·9	0·07	19·0	0·30	4·5	0·05	13·1	0·26	4·4	0·04	10·7	0·17	2·7	0·03	7·9	0·16
	F	Northern Europe	6·1	0·08	16·2	0·33	4·6	0·08	12·4	0·34	4·2	0·06	10·6	0·20	2·2	0·04	5·9	0·16
	D	Eastern Europe	6·2	0·05	19·1	0·23	3·8	0·04	12·2	0·16	5·9	0·04	15·3	0·15	0·8	0·01	2·7	0·04
	G	Asia†	3·5	0·02	9·1	0·06	1·5	0·01	4·3	0·06	5·8	0·03	13·4	0·11	0·2	0·00	0·6	0·01
	L	Asia†	6·5	0·04	15·6	0·15	1·6	0·01	4·3	0·06	5·8	0·04	11·6	0·14	2·1	0·02	5·5	0·07
	C	Northern Africa/Middle East	2·6	0·03	9·1	0·20	2·9	0·05	11·5	0·33	4·7	0·04	11·0	0·18	0·3	0·01	1·3	0·04
	A	Central Africa‡	2·3	0·05	7·3	0·12	0·8	0·02	2·5	0·06	4·9	0·12	12·8	0·25	0·1	0·00	0·4	0·01
	J	Central Africa‡	1·0	0·01	4·2	0·04	0·7	0·01	4·1	0·07	5·1	0·06	18·0	0·30	0·1	0·00	0·4	0·01
	I	Southern Africa	3·5	0·03	10·7	0·08	2·1	0·02	7·9	0·08	5·7	0·04	11·4	0·11	0·2	0·00	0·8	0·01

* Geographic diet clusters were based on the 2006 Global Environment Monitoring System (GEMS)/Food Contamination Monitoring and Assessment Programme (GEMS/Food) clusters and the 2002–7 FAO supply utilisation accounts data; F&V intakes were as reported in the 2002–4 WHS data; flavonoid concentration data were obtained from the US Department of Agriculture Flavonoid Database, 3.1; ellagic acid concentration data were obtained from the published literature.

† Asia was separated by the GEMS into two clusters; both diets were high in rice and wheat. Cluster G was characterised by higher availability of fruiting vegetables, milk and milk products, potatoes, and fish/seafood and fish/seafood products, while cluster L was characterised by higher availability of fish/seafood and fish/seafood products, maize, milk and milk products, and brassica vegetables.

‡ Central Africa was separated by the GEMS into two clusters. Cluster A was characterised by higher availability of plantains, cassava, rice, wheat, maize, and milk and milk products. Cluster J was characterised by higher availability of cassava, sorghum, milk and milk products, millet, rice, and maize.

### Contributions to phytonutrient intakes by type of fruits and vegetables and geographic diet cluster

The top three ranked contributions to the estimated phytonutrient intakes by source (limited to sources accounting for ≥ 3 % of the estimated phytonutrient intakes) are presented in Table 4 (carotenoids) and Table 5 (flavonoids and ellagic acid). For four of the five carotenoids (α-carotene, β-carotene, lutein/zeaxanthin and lycopene), a single vegetable category ranked as the top source of the phytonutrients in nine or more of the thirteen geographic diet clusters. ‘Oranges’ were the top source of hesperetin in twelve geographic diet clusters, and ‘Onions, dry’ accounted for the largest proportion of quercetin intake in all geographic diet clusters.

**Table 4:** Top fruit and vegetable sources of carotenoids (μg/d)* in the 2002–4 World Health Survey (WHS) assessed by geographic diet cluster

Geographic diet cluster†	α-Carotene (%)	β-Carotene (%)	β-Cryptoxanthin (%)	Lutein/zeaxanthin (%)	Lycopene (%)
M	Americas and Australia	Carrots and turnips (68 %), Pumpkins, squash and gourds (11 %), Tomatoes (8 %)	Carrots and turnips (28 %), Lettuce and chicory (25 %), Tomatoes (11 %)	Oranges (30 %), Tangerines, mandarins, clementines (19 %), Orange juice (13 %)	Lettuce and chicory (36 %), Cabbages and other brassicas (17 %), Spinach (10 %)	Tomatoes (73 %), Watermelons (21 %)
H	South/Central America	Carrots and turnips (54 %), Plantains (21 %), Pumpkins, squash and gourds (11 %)	Carrots and turnips (32 %), Tomatoes (9 %), Lettuce and chicory (9 %)	Papayas (32 %), Oranges (28 %), Tangerines, mandarins, clementines (11 %)	Cabbages and other brassicas (19 %), Lettuce and chicory (16 %), Maize (10 %)	Tomatoes (48 %), Mangoes, mangosteens, guavas (23 %), Watermelons (19 %)
K	South America	Carrots and turnips (50 %), Plantains (20 %), Tomatoes (12 %)	Carrots and turnips (31 %), Tomatoes (24 %), Cabbages and other brassicas (12 %)	Papayas (37 %), Oranges (24 %), Tangerines, mandarins, clementines (18 %)	Cabbages and other brassicas (37 %), Tomatoes (13 %), Pumpkins, squash and gourds (8 %)	Tomatoes (75 %), Watermelons (13 %), Mangoes, mangosteens, guavas (6 %)
B	Southern Europe/Mediterranean	Carrots and turnips (62 %), Tomatoes (13 %), Pumpkins, squash and gourds (9 %)	Carrots and turnips (23 %), Tomatoes (15 %), Lettuce and chicory (13 %)	Tangerines, mandarins, clementines (29 %), Oranges (25 %), Watermelons (11 %)	Lettuce and chicory (22 %), Cabbages and other brassicas (21 %), Spinach (16 %)	Tomatoes (63 %), Watermelons (34 %)
E	Western Europe	Carrots and turnips (82 %), Veg, dehydrated (4 %), Pumpkins, squash and gourds (3 %)	Carrots and turnips (42 %), Cabbages and other brassicas (16 %), Lettuce and chicory (9 %)	Tangerines, mandarins, clementines (33 %), Orange juice (17 %), Oranges (11 %)	Cabbages and other brassicas (35 %), Lettuce and chicory (14 %), Veg, frozen (10 %)	Tomatoes (66 %), Watermelons (18 %), Veg, dehydrated (5 %)
F	Northern Europe	Carrots and turnips (88 %), Veg, dehydrated (3 %)	Carrots and turnips (51 %), Cabbages and other brassicas (19 %), Lettuce and chicory (7 %)	Tangerines, mandarins, clementines (35 %), Orange juice (18 %), Oranges (13 %)	Cabbages and other brassicas (47 %), Lettuce and chicory (12 %), Veg, prepared (11 %)	Tomatoes (56 %), Watermelons (23 %), Tomato juice (11 %)
D	Eastern Europe	Carrots and turnips (76 %), Pumpkins, squash and gourds (12 %), Tomatoes (6 %)	Carrots and turnips (38 %), Cabbages and other brassicas (32 %), Tomatoes (10 %)	Tangerines, mandarins, clementines (24 %), Oranges (17 %), Watermelons (16 %)	Cabbages and other brassicas (73 %), Pumpkins, squash and gourds (7 %), Tomatoes (4 %)	Tomatoes (62 %), Watermelons (37 %)
G	Asia‡	Carrots and turnips (62 %), Pumpkins, squash and gourds (18 %), Tomatoes (6 %)	Cabbages and other brassicas (28 %), Spinach (20 %), Carrots and turnips (15 %)	Tangerines, mandarins, clementines (23 %), Watermelons (19 %), Persimmons (15 %)	Cabbages and other brassicas (41 %), Spinach (33 %), Lettuce and chicory (10 %)	Watermelons (54 %), Tomatoes (32 %), Mangoes, mangosteens, guavas (12 %)
L	Asia‡	Carrots and turnips (60 %), Pumpkins, squash and gourds (14 %), Veg, dehydrated (8 %)	Cabbages and other brassicas (41 %), Carrots and turnips (17 %), Spinach (7 %)	Persimmons (40 %), Tangerines, mandarins, clementines (30 %), Papayas (5 %)	Cabbages and other brassicas (60 %), Spinach (12 %), Lettuce and chicory (6 %)	Watermelons (44 %), Tomatoes (23 %), Mangoes, mangosteens, guavas (21 %)
C	Northern Africa/Middle East	Carrots and turnips (49 %), Pumpkins, squash and gourds (19 %), Tomatoes (15 %)	Carrots and turnips (23 %), Tomatoes (23 %), Cabbages and other brassicas (9 %)	Tangerines, mandarins, clementines (25 %), Oranges (21 %), Watermelons (19 %)	Cabbages and other brassicas (23 %), Pumpkins, squash and gourds (13 %), Tomatoes (10 %)	Tomatoes (71 %), Watermelons (26 %)
A	Central Africa§	Plantains (63 %), Pumpkins, squash and gourds (19 %), Carrots and turnips (8 %)	Plantains (29 %), Cabbages and other brassicas (19 %), Pumpkins, squash and gourds (14 %)	Maize (46 %), Papayas (24 %), Chillies and peppers, green (9 %)	Cabbages and other brassicas (38 %), Pumpkins, squash and gourds (20 %), Maize (19 %)	Tomatoes (72 %), Mangoes, mangosteens, guavas (16 %), Watermelons (6 %)
J	Central Africa§	Plantains (48 %), Carrots and turnips (38 %), Tomatoes (5 %)	Carrots and turnips (27 %), Plantains (16 %), Tomatoes (11 %)	Papayas (62 %), Maize (14 %), Chillies and peppers, green (12 %)	Cabbages and other brassicas (26 %), Maize (17 %), Okra (13 %)	Tomatoes (43 %), Mangoes, mangosteens, guavas (23 %), Watermelons (21 %)
I	Southern Africa	Plantains (46 %), Carrots and turnips (32 %), Pumpkins, squash and gourds (8 %)	Cabbages and other brassicas (38 %), Carrots and turnips (17 %), Plantains (11 %)	Maize (25 %), Oranges (20 %), Chillies and peppers, green (18 %)	Cabbages and other brassicas (66 %), Spinach (6 %), Maize (5 %)	Tomatoes (74 %), Mangoes, mangosteens, guavas (16 %), Watermelons (3 %)

Veg, vegetables.

* Percentage contribution by fruit and vegetable categories contributing ≥ 3 %, in descending order.

† Geographic diet clusters were based on the 2006 Global Environment Monitoring System (GEMS)/Food Contamination Monitoring and Assessment Programme (GEMS/Food) clusters and the 2002–7 FAO supply utilisation accounts data; fruit and vegetable intakes were as reported in the 2002–4 WHS data; carotenoid concentration data were obtained from the US Department of Agriculture National Nutrient Database, Standard Reference Release 25.

‡ Asia was separated by the GEMS into two clusters; both diets were high in rice and wheat. Cluster G was characterised by higher availability of fruiting vegetables, milk and milk products, potatoes, and fish/seafood and fish/seafood products, while cluster L was characterised by higher availability of fish/seafood and fish/seafood products, maize, milk and milk products, and brassica vegetables.

§ Central Africa was separated by the GEMS into two clusters. Cluster A was characterised by higher availability of plantains, cassava, rice, wheat, maize, and milk and milk products. Cluster J was characterised by higher availability of cassava, sorghum, milk and milk products, millet, rice, and maize.

**Table 5:** Top fruit and vegetable sources of select flavonoids and ellagic acid (mg/d)* in the 2002–4 World Health Survey (WHS) assessed by geographic diet cluster

Geographic diet cluster†	Anthocyanidins (%)	Hesperetin (%)	Quercetin (%)	Ellagic acid (%)
M	Americas and Australia	Lettuce and chicory (24 %), Bananas (12 %), Cranberries (12 %)	Oranges (61 %), Lemons and limes (20 %), Orange juice (14 %)	Onions, dry (46 %), Lettuce and chicory (16 %), Chillies and peppers, green (8 %)	Strawberries (72 %), Raspberries (18 %), Apples (3 %)
H	South/Central America	Bananas (39 %), Lettuce and chicory (16 %), Onions, dry (14 %)	Oranges (67 %), Lemons and limes (30 %)	Onions, dry (56 %), Chillies and peppers, green (22 %), Lettuce and chicory (4 %)	Strawberries (70 %), Bananas (10 %), Raspberries (7 %)
K	South America	Bananas (54 %), Onions, dry (21 %), Grapes (13 %)	Oranges (78 %), Lemons and limes (16 %), Tangerines, mandarins, clementines (5 %)	Onions, dry (76 %), Onions (inc. shallots), green (6 %), Tomatoes (6 %)	Bananas (38 %), Strawberries (30 %), Pineapples (18 %)
B	Southern Europe/Mediterranean	Grapes (48 %), Lettuce and chicory (14 %), Bananas (8 %)	Oranges (66 %), Lemons and limes (25 %), Tangerines, mandarins, clementines (6 %)	Onions, dry (42 %), Chillies and peppers, green (17 %), Apples (9 %)	Strawberries (79 %), Apples (7 %), Pears (3 %)
E	Western Europe	Grapes (17 %), Currants (16 %), Lettuce and chicory (13 %)	Oranges (42 %), Orange juice (25 %), Lemons and limes (19 %)	Onions, dry (45 %), Apples (13 %), Lettuce and chicory (9 %)	Strawberries (62 %), Raspberries (28 %), Apples (4 %)
F	Northern Europe	Bananas (20 %), Lettuce and chicory (16 %), Grapes (14 %)	Oranges (49 %), Orange juice (26 %), Lemons and limes (12 %)	Onions, dry (43 %), Lettuce and chicory (10 %), Apples (10 %)	Strawberries (73 %), Raspberries (14 %), Bananas (4 %)
D	Eastern Europe	Grapes (33 %), Currants (15 %), Onions, dry (9 %)	Oranges (64 %), Lemons and limes (26 %), Tangerines, mandarins, clementines (8 %)	Onions, dry (67 %), Apples (7 %), Cabbages and other brassicas (7 %)	Strawberries (79 %), Apples (7 %), Pears (3 %)
G	Asia‡	Lettuce and chicory (26 %), Bananas (24 %), Cabbages and other brassicas (14 %)	Oranges (59 %), Tangerines, mandarins, clementines (22 %), Lemons and limes (17 %)	Onions, dry (52 %), Chillies and peppers, green (11 %), Cabbages and other brassicas (7 %)	Bananas (27 %), Strawberries (22 %), Apples (14 %)
L	Asia‡	Bananas (43 %), Strawberries (12 %), Cabbages and other brassicas (12 %)	Tangerines, mandarins, clementines (45 %), Oranges (28 %), Orange juice (11 %)	Onions, dry (52 %), Cabbages and other brassicas (12 %), Onions (inc. shallots), green (11 %)	Strawberries (81 %), Bananas (9 %), Tangerines, mandarins, clementines (3 %)
C	Northern Africa/Middle East	Grapes (49 %), Onions, dry (15 %), Bananas (10 %)	Oranges (72 %), Lemons and limes (16 %), Tangerines, mandarins, clementines (7 %)	Onions, dry (61 %), Chillies and peppers, green (12 %), Tomatoes (7 %)	Strawberries (81 %), Apples (6 %), Bananas (5 %)
A	Central Africa§	Bananas (64 %), Onions, dry (20 %), Grapes (8 %)	Oranges (79 %), Lemons and limes (15 %)	Onions, dry (72 %), Chillies and peppers, green (9 %), Okra (5 %)	Bananas (84 %), Pineapples (10 %)
J	Central Africa§	Onions, dry (41 %), Bananas (36 %), Lettuce and chicory (11 %)	Oranges (69 %), Lemons and limes (27 %)	Onions, dry (59 %), Chillies and peppers, green (16 %), Okra (12 %)	Pineapples (70 %), Bananas (27 %)
I	Southern Africa	Bananas (63 %), Cabbages and other brassicas (14 %), Onions, dry (12 %)	Oranges (85 %), Orange juice (6 %), Lemons and limes (5 %)	Onions, dry (60 %), Chillies and peppers, green (16 %), Cabbages and other brassicas (9 %)	Bananas (65 %), Pineapples (10 %), Strawberries (10 %)

inc., Including.

* Percentage contribution by fruit and vegetable categories contributing ≥ 3 %, in descending order.

† Geographic diet clusters were based on the 2006 Global Environment Monitoring System (GEMS)/Food Contamination Monitoring and Assessment Programme (GEMS/Food) clusters and the 2002–7 FAO supply utilisation accounts data; fruit and vegetable intakes were as reported in the 2002–4 WHS data; flavonoid concentration data were obtained from the US Department of Agriculture Flavonoid Database, 3.1; ellagic acid concentration data were obtained from the published literature.

‡ Asia was separated by the GEMS into two clusters; both diets were high in rice and wheat. Cluster G was characterised by higher availability of fruiting vegetables, milk and milk products, potatoes, and fish/seafood and fish/seafood products, while cluster L was characterised by higher availability of fish/seafood and fish/seafood products, maize, milk and milk products, and brassica vegetables.

§ Central Africa was separated by the GEMS into two clusters. Cluster A was characterised by higher availability of plantains, cassava, rice, wheat, maize, and milk and milk products. Cluster J was characterised by higher availability of cassava, sorghum, milk and milk products, millet, rice, and maize.

## Discussion

In the present study, we estimated phytonutrient intakes by adults in thirteen geographical regions throughout the world. The estimates were generated by level of fruit and vegetable consumption ( < 5 *v.* ≥ 5 servings/d). By combining fruit and vegetable servings intake data at the individual level (WHS) with data on availability of specific fruits and vegetables in each geographic diet cluster (WHO/FAO), we approximated the intakes of specific categories of fruits and vegetables by level of fruit and vegetable consumption. These estimates of fruit and vegetable intakes were, in turn, combined with a large and systematically compiled database of phytonutrient concentration data to develop the estimates of phytonutrient intakes from fruit and vegetable sources for all adults by level of fruit and vegetable consumption. The estimates developed using this novel approach are clearly approximate rather than precise estimates of phytonutrient intakes. Nonetheless, given that the WHS and WHO/FAO data were collected in all countries using standardised methods and phytonutrient concentrations were assigned using a single database of values, the approximate phytonutrient intakes generated in the present study can be used to study the major patterns of phytonutrient intakes across geographical regions worldwide.

The results from the present analysis show that the estimated intakes of nine phytonutrients from fruit and vegetable sources varied by geographic diet cluster. As expected, adults with low fruit and vegetable consumption ( < 5 servings/d) had lower phytonutrient intakes than those consuming ≥ 5 servings/d of fruits and vegetables. The variations in estimated phytonutrient intakes observed across the geographic diet clusters reflect regional differences in both numbers and proportions of fruit and vegetable servings consumed, and the specific types of fruits and vegetables available in the diet. Overall, adults with low intakes of fruits and vegetables were estimated to consume approximately one-half to one-sixth the levels of phytonutrients consumed by adults with intakes of ≥ 5 servings/d of fruits and vegetables. The relative differences in phytonutrient intakes by level of fruit and vegetable consumption were comparable to findings in our previous assessment of intake by adults in the USA^(^ ^33^ ^)^, and generally similar to the results reported by Lee *et al.* ^(^ ^32^ ^)^ for adults in Korea.

In subpopulations of adults by level of fruit and vegetable consumption ( < 5 or ≥ 5 servings/d), the mean combined and individual intakes of fruits and vegetables differed by less than a factor of two across the geographic diet clusters. In contrast to the generally similar fruit and vegetable intakes observed among adults with low combined fruit and vegetable consumption, daily intakes of fruit servings by the subpopulation of men and women consuming ≥ 5 servings/d of fruits and vegetables exceeded the intakes of vegetable servings in a majority of the geographic diet clusters. These findings suggest that in addition to the expected differences in the number of fruit and vegetable servings consumed daily by adults with intakes of ≥ 5 servings of fruits and vegetables combined compared with those with low intakes, the proportion of total servings consumed as fruits (or vegetables) also differs, which would, in turn, have an impact on the differences in phytonutrient intakes.

The WHS included primarily low- and middle-income countries. In a previous analysis of the WHS data, prevalence of low fruit and vegetable consumption among the WHS countries differed by income within some, though not all, countries, and prevalence of low fruit and vegetable consumption overall was observed to be in the range reported for higher-income countries^(^ ^20^ ^)^. Higher income has been associated not only with an increase in the quantity of fruits and vegetables consumed, but also with increased variety^(^ ^62^ ^,^ ^63^ ^)^. Low-income countries, and particularly rural areas, struggle additionally with the fact that fruits and vegetables are highly perishable and technologies are not abundant to either store or preserve fresh produce to increase its availability^(^ ^64^ ^)^. All of these factors taken together could lead to lower intakes of both fruits and vegetables and their associated phytonutrients among those living in poor countries and/or rural areas.

Based on the reported availability of fruits and vegetables in each of the thirteen geographic diet clusters, there were apparent differences in the types of fruits and vegetables consumed in different geographical regions. The proportion of total fruits accounted for by tropical/subtropical fruits (items such as bananas, plantains, pineapples, papayas and mangoes) in each geographic diet cluster differed the most dramatically, with amounts ranging from 8·5 % in the region covering Eastern Europe to 93 % in the region spanning parts of Central Africa. Citrus fruits, pome fruits and watermelons were among the most consistently available fruits across most geographic diet clusters. Fruiting vegetables (excluding cucurbits) and mushrooms, a category that included items such as tomatoes, maize, eggplant and mushrooms, accounted for the greatest proportion of total vegetable intakes in most, though not all, geographic diet clusters. Brassica vegetables, bulb vegetables and fruiting vegetables including cucurbits were among the most consistently available vegetables across the geographic diet clusters. Overall, the proportion of total fruits or vegetables accounted for by many specific types differed by a factor of two or more across the geographic diet clusters. In contrast, relatively modest differences were observed in combined intakes of fruit and vegetable servings. In this analysis, differences in some types of fruits and vegetables available across the geographical regions appear to have a greater influence on total phytonutrient intakes than differences in amounts of fruits and vegetables consumed.

As expected, within each geographic diet cluster, adults with low fruit and vegetable consumption had lower intakes of phytonutrients than those consuming ≥ 5 servings/d of fruits and vegetables. We also observed some distinct patterns in the estimated intakes of phytonutrients from fruits and vegetables by geographical region. For example, the European regions, in particular Northern Europe, had comparatively high intakes of α-carotene and β-carotene; these intakes are probably attributable to high intakes of the ‘Carrots and turnips’ category of vegetables, as carrots are a concentrated source of these carotenoids. Intakes of ellagic acid were generally low in African regions, presumably as a result of limited availability of berries that are concentrated sources of this phytonutrient.

An interesting observation in the present study was that the estimated mean phytonutrient intakes by adults consuming ≥ 5 servings/d of fruits and vegetables in one geographic diet cluster were in some cases comparable to or lower than the mean intakes by adults with low fruit and vegetable consumption in another cluster. For example, the estimated daily intake of lutein/zeaxanthin by men in South/Central America reporting daily intakes of ≥ 5 servings of fruits and vegetables was 1286 μg. This level of intake was comparable to the mean daily intake of 1261 μg lutein/zeaxanthin among men in Western Europe identified as consuming low levels of fruits and vegetables. The large difference in lutein/zeaxanthin intake across these geographic diet clusters is probably attributable to the fact that brassica vegetables (typically a concentrated source of lutein/zeaxanthin) accounted for more than twice the proportion of total vegetables consumed by individuals in Western Europe compared with adults consuming a South/Central American diet. Substantial differences in the estimated intakes of phytonutrients across geographic diet clusters are worthy of further study in the context of health issues. It is important to consider, however, that our assessment included phytonutrient intake only from fruit and vegetable sources. Other plant-based foods such as tea, nuts, seeds, legumes and wine could also contribute to dietary intakes of some of the phytonutrients included in the present analysis.

Ranked contributions of each category of fruits and vegetables to total intakes of each phytonutrient were also estimated. Despite some notable differences in the relative availability of specific fruit and vegetable categories across the geographic diet clusters, there was considerable consistency in the top fruit and vegetable source categories for the phytonutrients included in the present analysis. This consistency across the regions may in part be attributed to the fact that some phytonutrients are found in a limited number of foods (e.g. lycopene in tomatoes, watermelon, guava and pink/red grapefruit), or that the phytonutrient occurs in many foods, but at concentrated levels in a relatively small number of commonly consumed foods (e.g. α-carotene in carrots). Bananas appeared among the top sources for both anthocyanidins and ellagic acid in some regions. This was a somewhat unexpected finding given the low levels of these phytonutrients in bananas relative to other sources (e.g. red or purple grapes and strawberries, respectively), though the results are not surprising given the large percentage of total fruits consumed as bananas in some geographic diet clusters. It is important to consider that not all fruits and vegetables found in all typical diets throughout the world were captured in the specific categories of fruits and vegetables included in the present analysis. Some less widely consumed foods may in fact be concentrated sources of one or more of the phytonutrients of interest, but if the food was not named in a FAO food category, our estimates of phytonutrient intakes would not reflect these contributions. For example, pomegranates are a concentrated source of ellagic acid relative to many more commonly consumed fruits, but they are not included among the specific fruit categories in the present analysis^(^ ^43^ ^,^ ^49^ ^)^.

There are several strengths to the present study, including the standardised approach for approximating fruit and vegetable consumption and the use of a single large and systematically compiled database of phytonutrient concentration data. However, some limitations must be considered when reviewing the findings of the present study. The exclusion of individuals with high intakes of fruits and vegetables (above the 99th percentile of intake) may have attenuated differences in phytonutrient intakes between the subpopulation of adults with low consumption of fruits and vegetables and those consuming ≥ 5 servings/d. As previously noted, the analysis was restricted to the specific categories of fruits and specific categories of vegetables corresponding to the FAO categories. In some cases, fruit and vegetable categories included the descriptor ‘nes’. In our analysis, intakes of the ‘nes’ categories were proportionally assigned to known categories of intake rather than additional types of fruits and vegetables that may have been available in the regions, as we could not determine with certainty the specific additional types of fruits and vegetables that may have been available. The available data also did not allow us to assign proportions to specific foods within categories representing more than one type of fruit or vegetable, e.g. what proportion of the ‘Carrots and turnips’ category was carrots *v.* turnips. Within each geographic diet cluster, fruit and vegetable consumption was based on WHS data from a subsample of adults in primarily low- and middle-income countries, and all adults in a cluster were assumed to consume the same proportions of fruits and vegetables based on the food availability data. We assumed that adults consuming a higher number of fruit and vegetable servings consumed larger quantities of fruits and vegetables in the proportions identified for the geographic diet cluster. This assumption may or may not be true, as it is possible that the higher number of fruit and vegetable servings reflects consumption of additional types of fruits and vegetables (i.e. a greater variety of consumption). Also, we assumed a default serving size of 80 g for all types of fruits and vegetables, though actual serving sizes may be higher or lower depending on both the specific item and regional consumption practices.

With limited exceptions for carotenoids, our estimates of carotenoid and flavonoid intakes were based on values reported in the USDA flavonoid database. We matched each FAO fruit and vegetable category to phytonutrient concentration data for a representative food for single-item categories, and multiple items for categories representing more than one specific fruit or vegetable (e.g. ‘Cabbages and other brassicas’) from which we then calculated an average value. The specific fruits and vegetables represented in the USDA database may not necessarily represent the items consumed across all geographic diet clusters. Handling and preparation factors may have an additional impact on phytonutrient levels in foods. Other investigators have supplemented the USDA data with additional sources, though they acknowledged that the amount of data added from additional sources was small^(^ ^29^ ^,^ ^65^ ^)^.

### Conclusions

The results from the present analysis show that the estimated intakes of nine phytonutrients from fruit and vegetable sources varied by geographic diet cluster. As expected, adults within a geographic diet cluster with low fruit and vegetable consumption ( < 5 servings/d) had lower phytonutrient intakes than those consuming ≥ 5 servings/d of fruits and vegetables. The mean phytonutrient intakes by adults consuming ≥ 5 servings/d of fruits and vegetables were approximately 2- to 6-fold the mean phytonutrient intakes by those with low fruit and vegetable consumption ( < 5 servings/d). The variations in phytonutrient intakes observed across the geographic diet clusters reflect regional differences in both numbers and proportions of fruit and vegetable servings consumed, and the specific types of fruits and vegetables available in the diet. The findings from this assessment provide important information regarding the major dietary patterns of phytonutrient intakes across the geographic diet clusters and can be used to direct further research to support the development of guidelines for intakes of phytonutrients.

## Supplementary material

To view supplementary material for this article, please visit http://dx.doi.org/10.1017/S0007114514001937
